# Integrating L-Cys-AuNCs in ZIF-8 with Enhanced Fluorescence and Strengthened Stability for Sensitive Detection of Copper Ions

**DOI:** 10.3390/molecules29246011

**Published:** 2024-12-20

**Authors:** Ting Zhou, Luyao Zang, Xia Zhang, Xia Liu, Zijie Qu, Guodong Zhang, Xiufeng Wang, Fang Wang, Zhiqing Zhang

**Affiliations:** Department of Chemistry, College of Chemistry and Chemical Engineering, China University of Petroleum (East China), Qingdao 266580, China

**Keywords:** fluorescence, L-Cys-AuNCs@ZIF-8, Cu^2+^ detection, quenching mechanism

## Abstract

Gold nanoclusters (AuNCs) have been widely investigated because of their unique photoluminescence properties. However, the applications of AuNCs are limited by their poor stability and relatively low fluorescence. In the present work, we developed nanocomposites (L-Cys-AuNCs@ZIF-8) with high fluorescence and stability, which were constructed by encapsulating the water-dispersible L-Cys-AuNCs into a ZIF-8 via Zn^2+^-triggered growth strategy without high temperature and pressure. The maximum emission wavelength of the L-Cys-AuNCs@ZIF-8 composite was at 868 nm, and the fluorescence intensity of L-Cys-AuNCs@ZIF-8 was nearly nine-fold compared with L-Cys-AuNCs without the ZIF-8 package. The mechanism investigation by fluorescence spectroscopy and X-ray photoelectron spectroscopy showed that L-Cys-AuNCs@ZIF-8 impeded ligand rotation, induced energy dissipation, and diminished the self-quenching effect, attributing to the spatial distribution of L-Cys-AuNCs. Based on the high fluorescence efficiency of L-Cys-AuNCs@ZIF-8, a “signal off” detective platform was proposed with copper ions as a model analyte, achieving a sensitive detection limit of Cu^2+^ at 16.7 nM. The quenching mechanism was confirmed, showing that the structure of the L-Cys-AuNCs@ZIF-8 nanocomposites was collapsed by the addition of Cu^2+^. Attributing to the strong adsorption ability between copper ions and pyridyl nitrogen, the as-prepared L-Cys-AuNCs@ZIF-8 was shown to accumulate Cu^2+^, and the Zn^2+^ in ZIF-8 was replaced by Cu^2+^.

## 1. Introduction

Gold nanoclusters (AuNCs), which consist of atoms, have achieved considerable attention in sensing, catalysis, diagnostic, and therapeutic applications due to their customizable structures and significant physical and chemical characteristics [1,2,3,4]. Ligands play a significant role in stabilizing and regulating the properties of AuNCs [2,5,6]. Ligands consisting of amino acids with rich sulfhydryl groups, such as bovine serum albumin (BSA), glutathione (GSH), and cysteine (Cys), can not only act as reducing agents and stabilizers to synthesize AuNCs, but also possess inherent biocompatibility and biodegradability [5,6]. However, one major drawback of the thiol-amino acid-capped AuNCs is their inadequate stabilization and relatively low luminescent efficiency [7,8]. Consequently, various research has focused on strategies to resolve this limitation by constructing composite systems, such as rigidifying nanoclusters by encapsulating them in polymer, metal oxide, or mesoporous supports [7,8,9,10,11]. Fu et al. have demonstrated the enhanced photo-luminescence intensity of AuNCs by poly (allyamine hydrochloride) through self-assembly strategies [9]. Hu et al. have reported the synthesis of extremely small gold nanoclusters encapsulated in hollow porous silica nanospheres for enhanced catalytic nitrophenol reduction [10]. Compositing of AuNCs and additional materials could exhibit improved stability or facilitate aggregation-induced emission (AIE) signals.

As a promising and emerging porous crystalline nanomaterial, metal-organic frameworks (MOFs) have obtained much attention owing to their inherent and distinctive structural features, including their atomically metallic sites, large specific surface areas, and porous construction [12,13]. Previous researchers have demonstrated that MOFs, with their excellent confinement effects, could be utilized for enhancing the properties of AuNCs [14,15,16,17,18]. Gao et al. have verified that the quantum yields and lifetime of AuNCs were greatly improved by embedding AuNCs in ZIF-8 via a crystalline ion-triggered growth strategy [15]. Jia et al. have reported that highly electroactive ZIF-67/PdNCs were used as an efficient co-reaction accelerator for improving the near-infrared electrochemiluminescence emission of NAC/Cys-AuNCs [16]. Researchers have constructed fluorescent sensing platforms by encapsulating GSH-AuNCs with an AIE effect into ZIF-8 for the sensitive detection of rutin or captopril [17,18]. Consequently, MOFs can restrict the rotation and vibration of the ligands of AuNCs to restrain nonradiative transition, and they can also reduce the self-quenching effect of AuNCs by spatial distribution to generate significantly improved fluorescence efficiency [14,17].

**Scheme 1:**
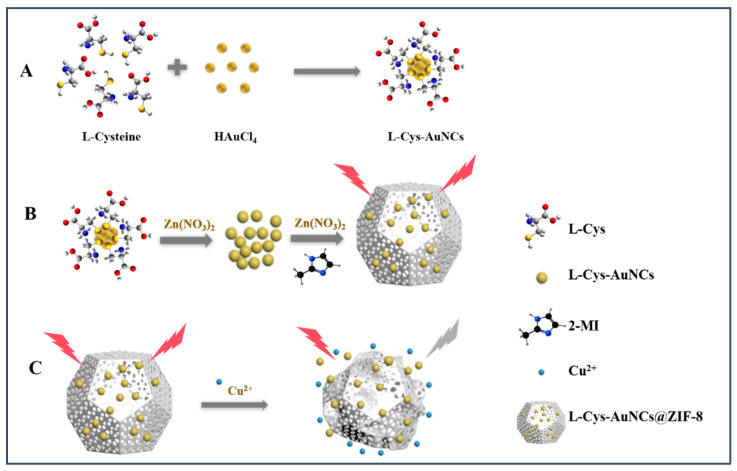
(**A**) Synthesis of L-Cys-AuNCs; (**B**) illustration showing the fabrication of L-Cys-AuNCs@ZIF-8 with improved fluorescence; (**C**) the as-prepared L-Cys-AuNCs@ZIF-8, used for Cu^2+^ detection.

Copper plays an indispensable role in pathological fields, and it is also vigorously used in various copper-based pesticide formulation [19]. In view of the notably detrimental effects that copper can have on human health and the environment, various strategies have been developed for Cu^2+^ detection [20,21,22,23,24,25,26,27,28,29], such as the efficient capture and detection of Cu^2+^ by all-in-one zinc-doped Prussian blue nanozyme [19], and an array of femtoliter wells for copper detection using click chemistry [29]. Integrating MOFs with nanomaterials as chemical receptors and transducers supplies benefits for copper ion detection [30,31,32,33,34], such as in a Co@ZIF-8 nanoparticle-decorated fiber optic sensor [30], or in MOFs in conjunction with fluorescent nanoclusters [33,34]. Moreover, the accumulation of MOFs can significantly enhance sensing ability, while maintaining stability and selectivity [14]. In the present work, we demonstrate the strategy of encapsulating L-Cys-AuNCs into metal organic frameworks (ZIF-8) to enhance fluorescence. Considering the strong adsorption ability of Cu^2+^ and imidazole, L-Cys-AuNCs@ZIF-8 with improved fluorescence could be used for the sensitive detection of Cu^2+^, as shown in Scheme 1. Briefly, the water-dispersible L-Cys-AuNCs were precipitated by Zn^2+^ and encapsulated into ZIF-8 by mixing with zinc nitrate and 2-methylimidazole. L-Cys-AuNCs@ZIF-8 was constructed with high fluorescence and stability through the aggregation-induced emission effect. The as-prepared L-Cys-AuNCs@ZIF-8 was shown to accumulate Cu^2+^, attributed to the strong adsorption ability between copper ions and pyridyl nitrogen, and the Zn^2+^ in the ZIF-8 was replaced by Cu^2+^, providing significant fluorescence quenching with a highly sensitive detection factor of 16.7 nM for Cu^2+^.

## 2. Results and Discussion

### 2.1. Synthesis and Characteristics of L-Cys-AuNCs@ZIF-8

**Figure 1:**
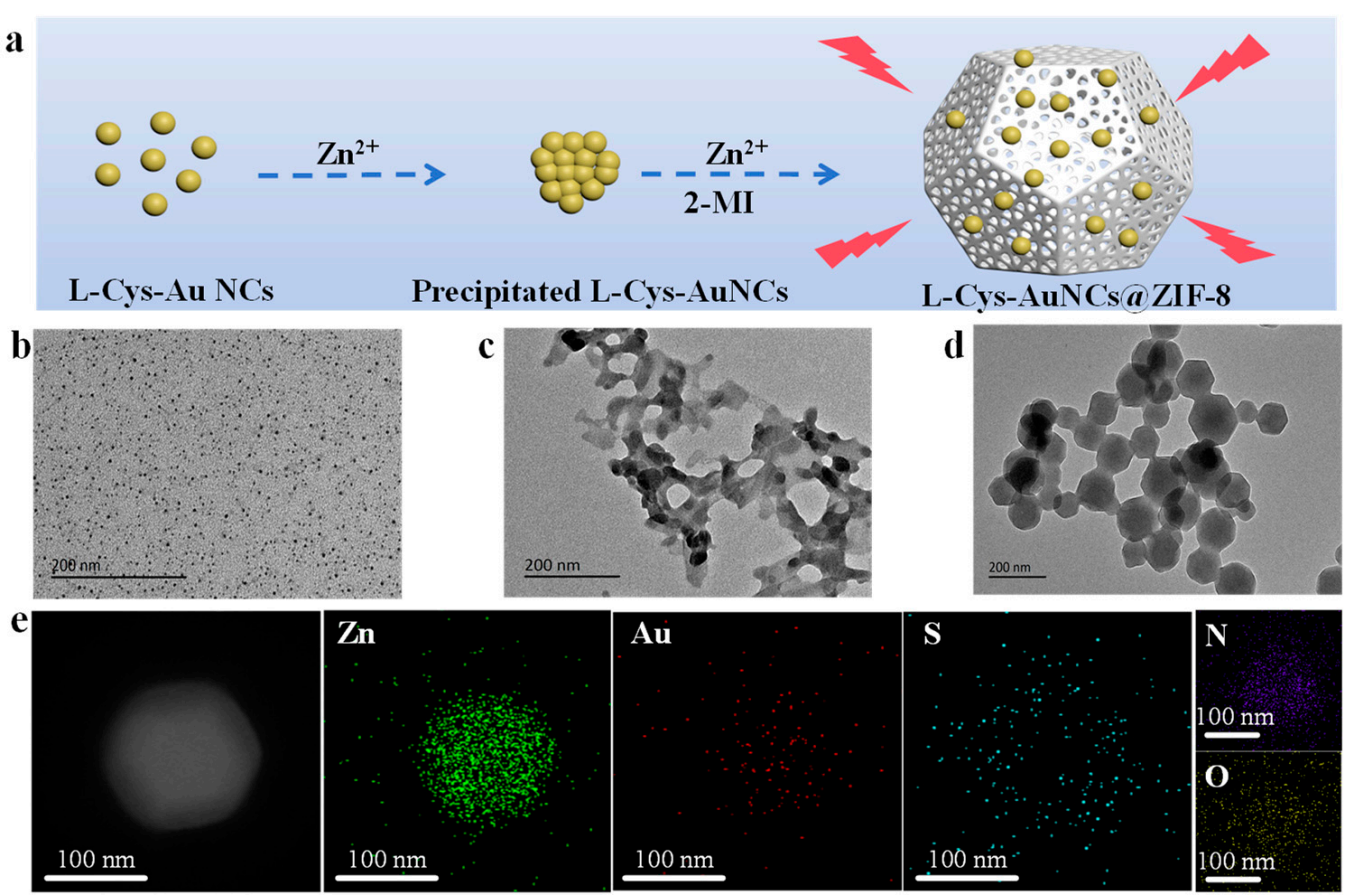
Schematic illustration for preparation of L-Cys-AuNCs@ZIF-8 (**a**); TEM images of L-Cys-AuNCs (**b**), the structure of L-Cys-AuNCs which was precipitated by Zn^2+^ (**c**) and L-Cys-AuNCs@ZIF-8 (**d**), respectively; high resolution transmission electron microscope image and the corresponding elemental (Zn, Au, S, N, O) mappings of L-Cys-AuNCs@ZIF-8 (**e**).

The schematic illustration for the synthetic strategy for L-Cys-AuNCs@ZIF-8 is shown in Figure 1a. In the synthesis protocol of L-Cys-AuNCs, L-cysteine (L-Cys) acted as a reducing agent and stabilizer, and HAuCl_4_ was reduced by the thiol group of L-Cys [35,36,37]. As revealed in Figure 1b, the obtained L-Cys-AuNCs were well monodispersed, with an average diameter of approximately 2.3 nm (Figure S1). The size of the L-Cys-AuNCs was relatively larger than the pores of ZIF-8 [17], and it was thus predictable that most of the L-Cys-AuNCs were inclined to integrate into ZIF-8. Subsequently, L-Cys-AuNCs were precipitated by adding Zn^2+^, and significant precipitation of L-Cys-AuNCs can be observed in Figure 1c. The precipitated L-Cys-AuNCs were then mixed with zinc hexahydrate and 2-methylimidazole (2-MI), achieving the encapsulation of L-Cys-AuNCs by the coordination of Zn^2+^ with the carboxyl group (the cysteine ligand of L-Cys-AuNCs) and the N atom in 2-methazole. The successful synthesis of L-Cys-AuNCs@ZIF-8 nanocomposites could be clearly observed through TEM imaging (Figure 1d). L-Cys-AuNCs@ZIF-8 had a uniform dodecahedral morphology with a statistical particle size of about 128 nm (Figure S2a). The hydrodynamic radius (*R*_H_) of L-Cys-AuNCs@ZIF-8 was obtained by dynamic light scattering (DLS) measurement and used to investigate the size distribution. The most probable *R_H_* value of the nanocomposites was 158 nm (Figure S2b), which was slightly larger than that of the statistical result from TEM due to the existence of the hydrolysis layer. Furthermore, the corresponding elemental mapping of L-Cys-AuNCs@ZIF-8 (Figure 1e) was performed. It could be clearly seen that each L-Cys-AuNCs@ZIF-8 nanoparticle contained N, O, and Zn elements, indicating the formation of the ZIF-8 skeleton. Meanwhile, the Au and S elements were evenly distributed in ZIF-8, confirming the encapsulation of L-Cys-AuNCs.

### 2.2. Investigation on Enhanced Luminescence of L-Cys-AuNCs@ZIF-8

**Figure 2:**
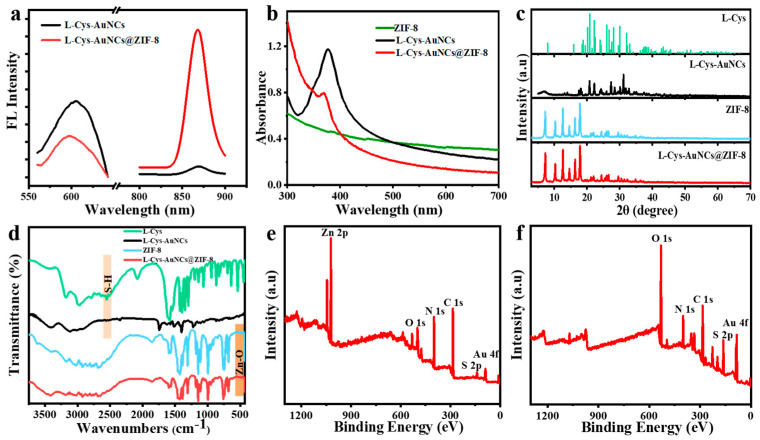
Fluorescence intensity of L-Cys-AuNCs and L-Cys-AuNCs@ZIF-8 (**a**); UV-vis spectra of ZIF-8, L-Cys-AuNCs, and L-Cys-AuNCs@ZIF-8 (**b**); X-ray diffraction (XRD) patterns of L-Cys, L-Cys-AuNCs, ZIF-8, and L-Cys-AuNCs@ZIF-8 (**c**); Fourier transform infrared (FT-IR) spectra of L-Cys, L-Cys-AuNCs, ZIF-8, and L-Cys-AuNCs@ZIF-8 (**d**); XPS of L-Cys-AuNCs@ZIF-8 (**e**) and L-Cys-AuNCs (**f**), respectively.

The fluorescence spectra of L-Cys-AuNCs and L-Cys-AuNCs@ZIF-8 were measured. The pristine L-Cys-AuNCs were shown to possess two emission peaks at 608 and 868 nm (Figure 2a). To acquire an optimal enhanced fluorescence performance of L-Cys-AuNCs@ZIF-8, the experimental condition exhibiting the optimum concentration of L-Cys-AuNCs reacting with ZIF-8 was investigated, and result is shown in Figure S3. As the concentration of L-Cys-AuNCs was increased, the emission fluorescence intensity at 868 nm firstly increased and reached a maximum value, and then decreased. Therefore, the AuNCs concentration was immobilized at 0.58 mM for synthesis of the following L-Cys-AuNCs@ZIF-8 nanomaterials. L-Cys-AuNCs@ZIF-8 exhibited fluorescence in the infrared region, with maximum intensity at around 868 nm. The intensity of these nanocomposites displayed a significant enhancement (almost nine-fold) compared to that of L-Cys-AuNCs (Figure 2a). The enhancement of fluorescence after ZIF-8 encapsulation was mainly due to the restriction of intramolecular movement of surface ligands [17]. Notably, the maximum emission of L-Cys-AuNCs at 608 nm was signally quenched (Figure 2a). This may be due to the significant effect of ZIF-8 on the metal charge transfer process of AuNCs through the confinement effect. The UV-vis absorption spectrum of L-Cys-AuNCs exhibited a distinct peak at 377 nm, whereas the spectra of the L-Cys ligand and ZIF-8 did not show any typical absorption peaks, as shown in Figure 2b and Figure S4. UV-vis spectra revealed a slightly blue-shift of the absorption peak from 377 nm for L-Cys-AuNCs to 368 nm for L-Cys-AuNCs@ZIF-8, which might have been due to the decrease in nanocluster size [38].

The successful construction of L-Cys-AuNCs@ZIF-8 was further confirmed by the wide-angle X-ray diffraction (XRD) pattern, Fourier transform infrared (FT-IR) spectra, and X-ray photoelectron spectra (XPS). The typical diffraction peaks of ZIF-8 are revealed in Figure 2c, and the XRD spectrum of L-Cys-AuNCs@ZIF-8 is consistent with that of pure ZIF-8, indicating that the coating of L-Cys-AuNCs does not affect the crystal growth of ZIF-8. Additionally, the FT-IR spectrum of L-Cys displays a wide but not deep band at 2555 cm^−1^, which is a characteristic absorption peak of the -SH stretching vibration [39]. As shown in Figure 2d, the disappearance of the -SH stretching vibration at 2555 cm^−1^ in L-Cys-AuNCs or L-Cys-AuNCs@ZIF-8 further proves that the ligand agents act on the surface of AuNCs through Au-S bonding [39]. The presence of a characteristic stretching vibration of Zn-N at 420 cm^−1^ (Figure 2d) indicates that the preparation of L-Cys-AuNCs@ZIF-8 depends on the coordination between the imidazole ring and Zn^2+^ [18]. According to the XPS spectrum of L-Cys-AuNCs@ ZIF-8, Au, S, Zn, O, and N elements were also observed in the prepared nanocomposites, further demonstrating the successful coating of L-Cys-AuNCs into ZIF-8 (Figure 2e,f).

**Figure 3:**
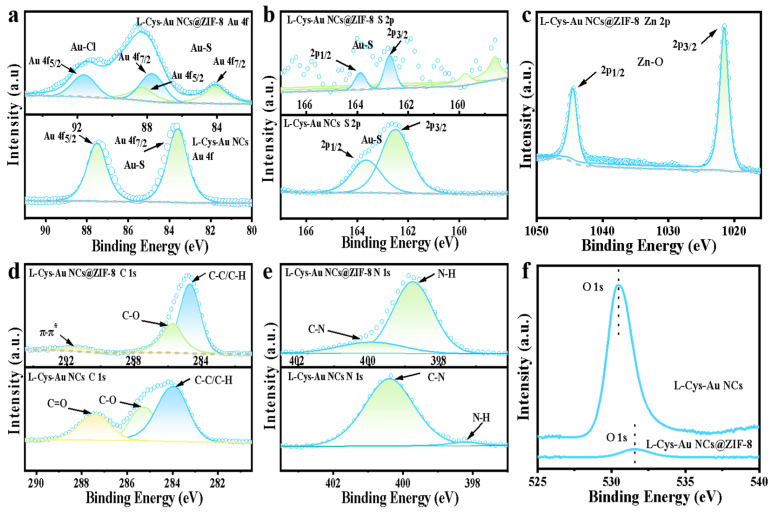
X-ray photoelectron spectra of Au (**a**), S (**b**), Zn (**c**), C (**d**), N (**e**), and O (**f**) in L-Cys-AuNCs@ZIF-8 and L-Cys-AuNCs, respectively.

Subsequently, X-ray photoelectron spectra were employed systematically to investigate how ligands interact with AuNCs, and to investigate the interaction between L-Cys-AuNCs and ZIF-8 (Figure 3). Au(I)–thiolate binding plays an important role in influencing the fluorescence properties of AuNCs, and the core 4f-level of nanoclusters was evaluated, as shown in Figure 3a. The binding energies of Au 4f7/2 in L-Cys-AuNCs@ZIF-8 and L-Cys-AuNCs were found to be 84.1 and 83.7 eV, respectively. Obviously, the binding energy in L-Cys-AuNCs@ZIF-8 shifted higher as compared with L-Cys-AuNCs, indicating that the electron density on the surface of Au had decreased. The binding of S 2p3/2 was 162.5 eV, owing to the Au-S covalent bond formation (Figure 3b). The XPS spectrum of Zn 2p3/2 shows that the binding energy of Zn was at 1022 eV, consistent with the binding energy of Zn in the oxidation state, indicating the existence of a Zn-O bond (Figure 3c). Different peaks for C-C/C-H, C-N, and C=O originating from the L-Cys ligand were evidently obtained from the XPS spectra of C 1s and N 1s (Figure 3d,e). It is worth noting that the O 1s spectrum of L-Cys-AuNCs@ZIF-8 was slightly shifted compared with L-Cys-AuNCs, as shown in Figure 3f. O 1s was the main peak in L-Cys-AuNCs, and its relative content was higher. Overall, based on the above spectroscopy results, L-Cys-AuNCs@ZIF-8 nanocomposites were evidently successfully synthesized.

### 2.3. Improved Stability of L-Cys-AuNCs@ZIF-8 Investigation

**Figure 4:**
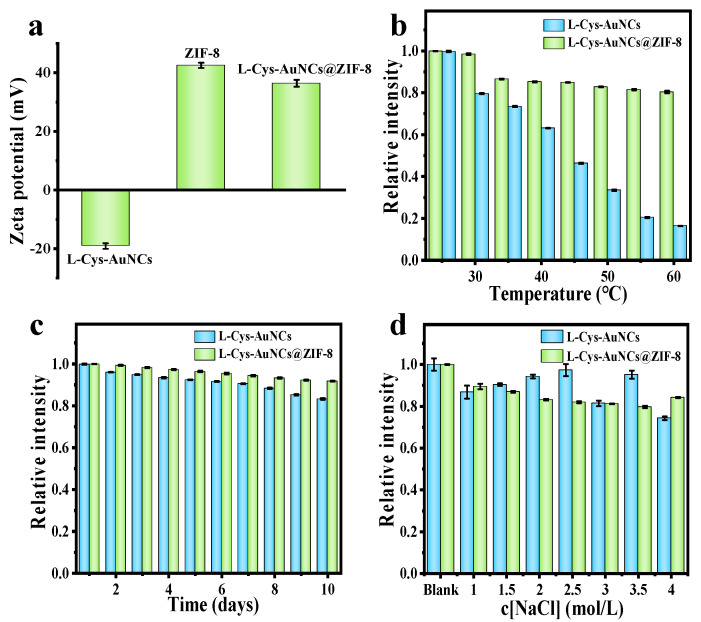
Zeta potential values of L-Cys-AuNCs, ZIF-8, and L-Cys-AuNCs@ZIF-8 (**a**); stability of L-Cys-AuNCs and L-Cys-AuNCs@ZIF-8 at different temperatures (**b**), after long-term storage (**c**), and at different concentrations of NaCl (**d**).

While the superior fluorescence of L-Cys-AuNCs@ZIF-8 is important in practical application fields, its stability is also crucial for its function and application. To investigate the stability of L-Cys-AuNCs@ZIF-8, we evaluated its tolerance to storage stability. Zeta potential is a valuable and important parameter used to describe the stability of nanomaterials [40]. As shown in Figure 4a, the zeta potential values of L-Cys-AuNCs and ZIF-8 were measured to be −19.1 mV and 42.5 mV, respectively, which are obviously opposite. The potential of L-Cys-AuNCs@ZIF-8 was 36.4 mV, indicating that the assembly of nanocomposites is achieved mainly through the electrostatic interaction between L-Cys-AuNCs and ZIF-8. Moreover, each particle is inevitably charged, and stability can be managed by the repulsion between different particles [40]. The relatively high zeta potential of L-Cys-AuNCs@ZIF-8 maintains the stability of system.

In opposition to the environmentally sensitive response exhibited by NCs [13,14,41], the fluorescence emissions of L-Cys-AuNCs@ZIF-8 were less sensitive to temperature changes as compared with L-Cys-AuNCs (Figure 4b). This can be further confirmed from the quantitative relationship between the emission intensities and temperature values: L-Cys-AuNCs@ZIF-8 showed high temperature independency at a wide range of temperatures (from 25~60 °C). Meanwhile, the emissions of L-Cys-AuNCs gradually reduced as temperature increases. At 60 °C, the emission quenching ratios of L-Cys-AuNCs@ZIF-8 and L-Cys-AuNCs were 20% and 84%, respectively. These results demonstrate that integrating L-Cys-AuNCs in ZIF-8 could increase their stability, showing excellent temperature-resistant properties. The fluorescence intensity of L-Cys-AuNCs@ZIF-8 was maintained at 92% after storage for 10 days, and the emission of L-Cys-AuNCs remained at 83% under the same conditions, as illustrated in Figure 4c. In addition, both of them maintain good fluorescence intensity under high salt concentrations (Figure 4d). In summary, ZIF-8-encapsulated L-Cys-AuNCs demonstrate an effective improvement in stability.

### 2.4. Sensitive Detection of Cu^2+^ by L-Cys-AuNCs@ZIF-8 and Possible Quenching Mechanism Investigation

**Figure 5:**
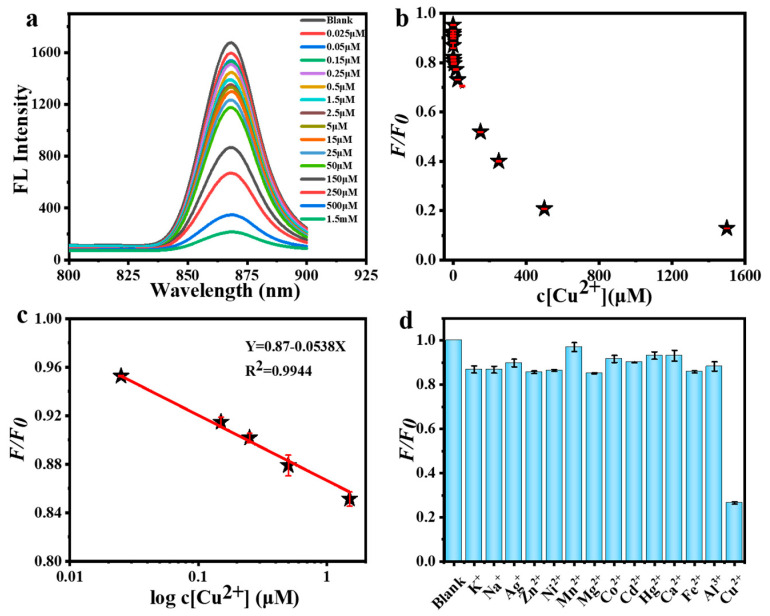
Fluorescence intensity evolution of L-Cys-AuNCs@ZIF-8 in the presence of different amounts of Cu^2+^ (**a**); *F*/*F*_0_ versus concentrations of Cu^2+^ (**b**); the linear relationship between *F*/*F*_0_ and Cu^2+^ at low concentrations (**c**); interference study with different metal ions (**d**).

L-Cys-AuNCs@ZIF-8 can be quenched by copper ions, showing excellent sensitivity and selectivity. Prior to the investigation on detecting copper ions, several factors which contribute to improving sensitivity were optimized, including the L-Cys-AuNCs@ZIF-8 concentration, pH value, and reaction time. As shown in Figure S5, the detection system showed desirable performance when the concentration of L-Cys-AuNCs@ZIF-8 was 2.6 mM, and the pH value was 9.0. In addition, the fluorescence ratio (*F*/*F*_0_) of the fluorescent probe (L-Cys-AuNCs@ZIF-8) remained balanced after incubating with Cu^2+^ for 1.0 min. Subsequently, the interaction between Cu^2+^ and L-Cys-AuNCs@ZIF-8 was systematically investigated. Under the optimal sensing conditions, the fluorescence spectra of L-Cys-AuNCs@ZIF-8 in the presence of different concentrations of Cu^2+^ were monitored, as illustrated in Figure 5a. L-Cys-AuNCs@ZIF-8 emission was progressively weakened as the concentration of Cu^2+^ increased from 25 nM to 1.5 mM. Moreover, the differences in emission intensity of L-Cys-AuNCs@ZIF-8 among each concentration of Cu^2+^ were relatively small, indicating the favorable stability of the detection system (Figure 5b). An excellent linear relationship between *F*/*F*_0_ and Cu^2+^ concentration is shown in Figure 5c, with a regression equation of y = −0.0538x + 0.87 (*R*^2^ = 0.9944). The linear range was 0.025–1.5 μM, and x was logc [Cu^2+^]. The limit of detection (LOD), calculated from 3σ/s, was 16.7 nM, which is lower than that reported in previous studies (Table 1) [20,22,24,25,26,27,34]. In addition, the reproducibility of the prepared L-Cys-AuNCs@ZIF-8 in the detection of Cu^2+^ was satisfactory, as shown in Figure S6. To further evaluate the selectivity for the detection of Cu^2+^, other species including K^+^, Ag^+^, Cd^2+^, Hg^2+^, Fe^2+^, Zn^2+^, etc. were selected as interfering substances. As Figure 5d shows, the presence of interfering metal ions resulted in negligible influence in L-Cys-AuNCs@ZIF-8 fluorescence. Results show that L-Cys-AuNCs@ZIF-8 is highly selective in distinguishing Cu^2+^ from other metal ions.

**Figure 6:**
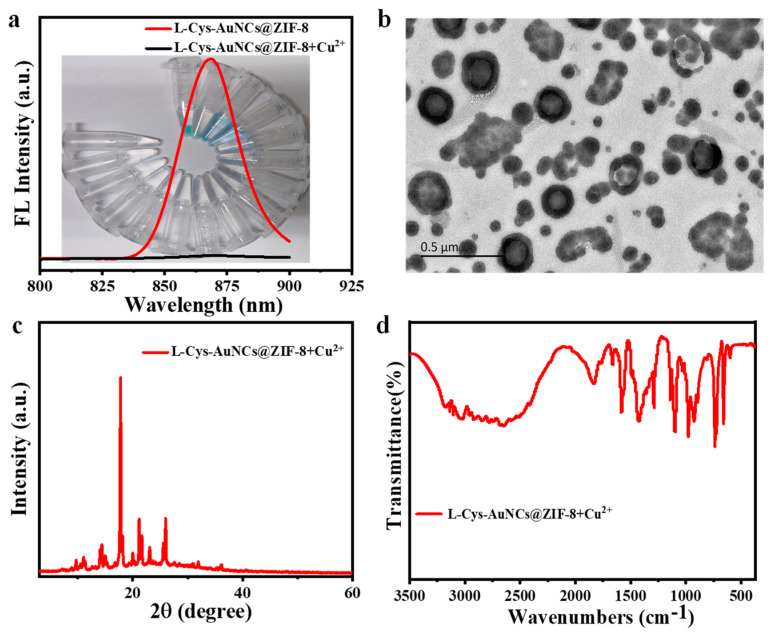
Fluorescence intensity and photograph of L-Cys-AuNCs@ZIF-8 and L-Cys-AuNCs@ZIF-8 + Cu^2+^, respectively (**a**); TEM image of L-Cys-AuNCs@ZIF-8 mixed with Cu^2+^ (**b**); XRD spectrum (**c**) and FT-IR spectrum (**d**) of L-Cys-AuNCs@ZIF-8 + Cu^2+^.

Subsequently, a series of experiments was performed to gain insight into the selective adsorption of Cu^2+^ ions by L-Cys-AuNCs@ZIF-8. Firstly, as shown in Figure 6a, the fluorescence of L-Cys-AuNCs@ZIF-8 can be effectively quenched after adding Cu^2+^, and blue precipitation was obviously observed after mixing L-Cys-AuNCs@ZIF-8 with higher concentrations of Cu^2+^. Moreover, after incubating with Cu^2+^, the original L-Cys-AuNCs@ZIF-8 with high crystallinity was transformed into low-crystallinity nanoparticles (Figure 1d and Figure 6b), consistent with the XRD pattern (Figure 6c). The diffraction peaks in the XRD spectrum of L-Cys-AuNCs@ZIF-8 almost disappeared after mixing with Cu^2+^, indicating the collapse of the ZIF-8 skeleton (Figure 2c and Figure 6c). This may be attributed to the fact that L-Cys-AuNCs@ZIF-8 is synthesized by the coordination of Zn^2+^ with the carboxyl group (cysteine) and nitrogen in 2-methylimidazole [34,42]. Attributing to the strong adsorption ability between copper ions and N atoms [20,22,24,25,26,27,34,42,43,44], Zn^2+^ in ZIF-8 was replaced by Cu^2+^, which might have triggered the decomposition of ZIF-8. The disappearance of the peak at 420 cm^−1^ (stretching vibration of Zn-N) in the FT-IR spectrum of L-Cys-AuNCs@ZIF-8 + Cu^2+^ further confirms that the frame structure of L-Cys-AuNCs@ZIF-8 changed greatly, as illustrated in Figure 2d and Figure 6d. The framework of L-Cys-AuNCs@ZIF-8 collapsed, accompanied by the release of L-Cys AuNCs with a very weak fluorescence signal at 868 nm. Therefore, the selective fluorescence quenching of L-Cys-AuNCs@ZIF-8 by copper ions over other metal ions was observed.

### 2.5. Application of L-Cys-AuNCs@ZIF-8 for Sensing Cu^2+^ in Practical Samples

In order to evaluate the application of the proposed sensing platform in actual samples, three water samples (tap water, river water, and seawater) containing different concentrations of copper ions (3, 5, 10 and 15 μM) were analyzed based on the standard calibration method. As Table 2 shows, the recoveries of the three samples were in the range of 94–125.9% (with RSD less than 1%), indicating that this method demonstrated relative feasibility for the detection of Cu^2+^ in actual samples.

## 3. Materials and Methods

### 3.1. Reagents

HAuCl_4_@4H_2_O, L-Cysteine (L-Cys), copper sulfate pentahydrate and 2-methylimidazole (2-MI), KCl, NaCl, AgNO_3_, NiSO_4_, MnCl_2_, MgCl_2_, CoCl_2_, CdCl_2_, HgCl_2_, CaCl_2_, FeCl_2_, and AlCl_3_ were purchased from Shanghai Aladdin Biochemical Technology Co., Ltd. (Shanghai, China). Zinc hexahydrate (Zn(NO_3_)_2_@6H_2_O) and AgNO_3_ were purchased from Sinopharm Chemical Reagent Co., Ltd. (Shanghai, China). Ultrapure water was obtained from MilliQ Instrument (Darmstadt, Germany). All glassware was washed with aqua regia and rinsed with ethanol and ultrapure water before use.

### 3.2. Preparation of L-Cys-AuNCs@ZIF-8

Based on the typical synthesis experiments for gold nanoclusters [35,36,37], HAuCl_4_ (20 mM, 100 μL) and L-Cys (100 mM, 300 μL) were added to ultrapure water (1.8 mL) under gentle stirring at 25 °C. When the light yellow solution became colorless, hydrochloric acid (200 μL, 1.0 M) was added. The mixture was heated to 40 °C under gentle stirring for 1.5 h, and L-Cys-AuNCs were obtained. Subsequently, 12 mL of L-Cys-AuNCs was precipitated by Zn(NO_3_)_2_·6H_2_O (960 μL 240 mM), and then the pH was adjusted to 2.0. The precipitates were centrifuged, and then dissolved in 4.0 mL of ultrapure water. The precipitated L-Cys-AuNCs were added dropwise to Zn(NO_3_)_2_·6H_2_O solution (0.2 g Zn(NO_3_)_2_·6H_2_O in 0.8 mL H_2_O), and then the mixture was dropwise added into 2-methylimidazole solution (2.0 g 2-methylimidazole in 8.0 mL H_2_O), and stirred for 10 min. Finally, opalescent L-Cys-AuNCs@ZIF-8 nanocomposites were refrigerated, washed three times with methanol, and then vacuum-dried overnight for subsequent use.

### 3.3. Fluorescence Detection of Cu^2+^

L-Cys-AuNCs@ZIF-8 was dispersed in ultrapure water for spectra measurement. Firstly, the optimized conditions for Cu^2+^ detection were selected. The concentrations of L-Cys-AuNCs@ZIF-8 were varied, and the pH values were changed from 1.0 to 12.0, variating the incubating time from 0.5 to 4.0 min. Under the optical detection conditions, 200 μL of copper sulfate solution at different concentrations was added to 200 μL of L-Cys-AuNCs@ZIF-8 solution (2.6 mM). After mixing for 1.0 min, the fluorescence emission intensities of L-Cys-AuNCs@ZIF-8 were measured as a function of Cu^2+^ concentrations, and the wavelength of excitation was 570 nm. The fluorescence spectra were measured in triplicate.

### 3.4. Selective Detection of Cu^2+^

For the selectivity experiments, different metal ions (K^+^, Na^+^, Ag^+^, Zn^2+^, Ni^2+^, Mn^2+^, Mg^2+^, Co^2+^, Cd^2+^, Hg^2+^, Ca^2+^, Fe^2+^, Al^3+^) were selected as the interference objects, and the fluorescence emission intensities of L-Cys-AuNCs@ZIF-8 (2.6 mM) mixed with different ions were measured at the excitation wavelength of 570 nm.

### 3.5. Detection of Cu^2+^ in Actual Water Samples

Three actual water samples of tap water, river water, and sea water were selected, and different concentrations of Cu^2+^ (3, 5, 10, 15 μM) were added to the water samples, following the standard addition method. The fluorescence detection methods used were the same as in Section 3.3.

## 4. Conclusions

In summary, L-Cys-AuNCs@ZIF-8 nanocomposites with improved luminescence and stability were constructed by integrating L-Cys-AuNCs into ZIF-8 for the sensitive detection of Cu^2+^. Benefiting from the coordination networks of ZIF-8, which restrict the rotation and vibration of the ligands of L-Cys-AuNCs and restrain the nonradiative transition, these nanocomposites demonstrated enhanced fluorescence properties and simultaneously exhibited greater stability. L-Cys-AuNCs@ZIF-8 was investigated for the sensitive and selective detection of Cu^2+^, which exhibited a wide range from 25 nM to 1.5 μM with a low limit detection of 16.7 nM for Cu^2+^ analysis. The as-proposed sensing platform was successfully evaluated for detecting Cu^2+^ in real water samples. Moreover, the mechanism of L-Cys-AuNCs@ZIF-8 fluorescence quenching by Cu^2+^ might be attributed to the synergistic effect of the collapse of the ZIF-8 framework, the strong interaction between Cu^2+^ and L-Cys, and the impeded Au-ligand charge transfer. Consequently, based on the aforementioned superiorities, L-Cys-AuNCs@ZIF-8 has exhibited a potential application in pollutant monitoring as a promising nanocomposite.

## Figures and Tables

**Table 1:** Comparison of different detection performances for Cu^2+^.

Nano Material	Linear Range (μM)	LOD (nM)	Ref.
CCYWDAHRDY-AuNCs	0.1–4.2	52	[27]
GS-AuNCs	0.1–6.25	86	[20]
BSA-protected GNCs	0.1–50	30	[22]
PTA-NH_2_@GSH-AuNCs	1–150	275	[26]
dNSiO_2_-AuAg NCs	0.2–2	60	[24]
GSH-AuNCs@ZIF-8	0–1.2	16	[34]
BSA-AuNCs	0–1724	830	[25]
L-Cys-AuNCs@ZIF-8	0.025–1.5	16.7	This work

**Table 2:** Recovery tests of Cu^2+^ in water samples.

Sample	Add (μM)	Found (μM)	Recovery (%)	RSD (%)
Tap water	3.0	2.82	94	0.45
	5.0	5.16	103.2	0.45
	10.0	9.53	95.3	0.47
	15.0	15.8	105.3	0.15
River water	3.0	3.31	110.3	0.41
	5.0	4.95	99.0	0.44
	10.0	11.05	110.5	0.12
	15.0	15.98	106.5	0.37
Sea water	3.0	2.86	95.3	0.5
	5.0	5.48	109.6	0.33
	10.0	12.59	125.9	0.26
	15.0	17.78	118.5	0.43

## Data Availability

Data are contained within the article and Supplementary Materials; further inquiries can be directed to the corresponding author.

## Supplementary Materials

The following supporting information can be downloaded at: https://www.mdpi.com/article/10.3390/molecules29246011/s1, Figure S1: Statistical diameter of L-Cys-Au NCs from TEM image; Figure S2: (a) Statistical diameter of L-Cys-Au NCs@ZIF-8 from TEM image. (b) Hydrodynamic radius of L-Cys-Au NCs@ZIF-8 measured by DLS; Figure S3: The different concentrations of L-Cys-Au NCs reacting with ZIF-8; Figure S4: UV-vis Spectrum of L-Cys; Figure S5: Optimization of L-Cys-Au NCs@ZIF-8 concentration (a); pH (b); and reaction time (c) with addition of copper ions alone; Figure S6: Reproducibility of Cu^2+^ detection.
