# Supplementary material for: Integrating L-Cys-AuNCs in ZIF-8 with Enhanced Fluorescence and Strengthened Stability for Sensitive Detection of Copper Ions
Source: Molecules. 2024 Dec 20;29(24):6011. doi: 10.3390/molecules29246011 (PMC11677756; doi:10.3390/molecules29246011)
Supplement: Supplementary file 1 [file molecules-29-06011-s001.zip › molecules-3345938-supplementary.pdf]

Supplementary material

Integrating L-Cys-AuNCs in ZIF-8 with Enhanced Fluorescence and Strengthened Stability for Sensitive Detection of Copper Ions

*Ting Zhou *, Luyao Zang, Xia Zhang, Xia Liu, Zijie Qu, Guodong Zhang, Xiufeng Wang, Fang Wang and Zhiqing Zhang **

Department of Chemistry, College of Chemistry and Chemical Engineering, China University of Petroleum (East China), Qingdao 266580, China

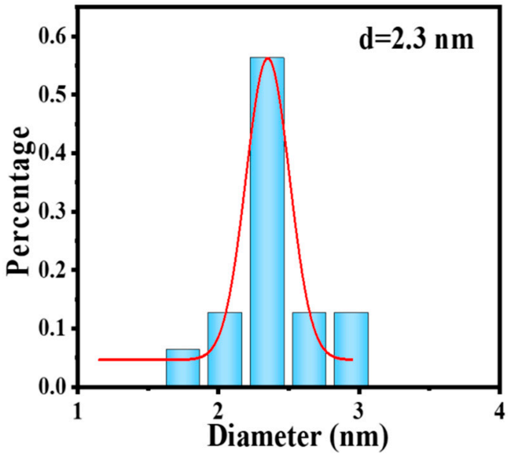

Figure S1. Statistical diameter of L-Cys-AuNCs from TEM image.

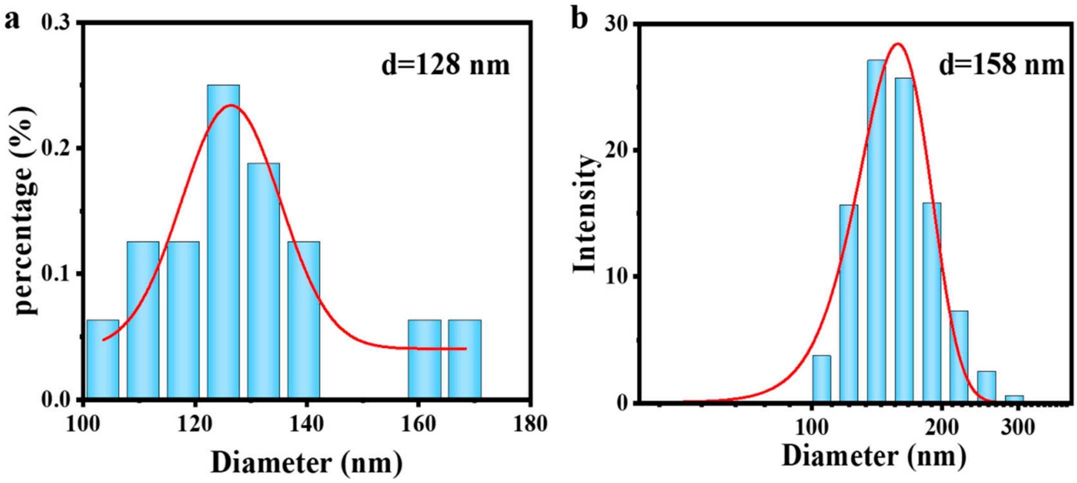

Figure S2. (a) Statistical diameter of L-Cys-AuNCs@ZIF-8 from TEM image.

(b) Hydrodynamic radius of L-Cys-AuNCs@ZIF-8 measured by DLS.

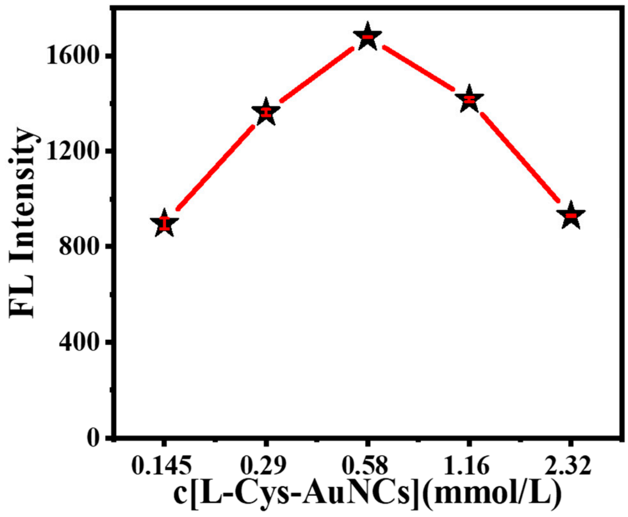

Figure S3. The different concentrations of L-Cys-AuNCs reacting with ZIF-8.

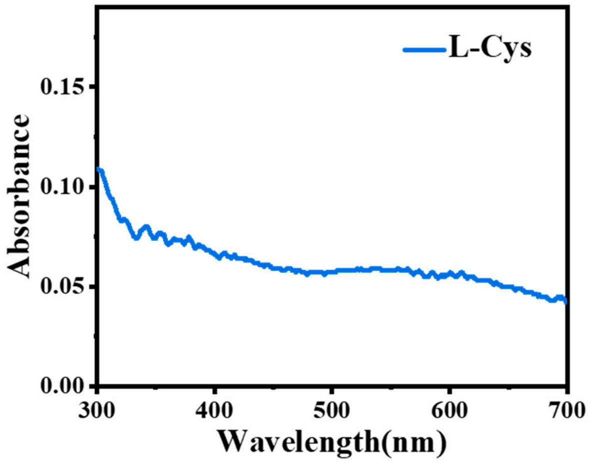

Figure S4. UV-*vis* Spectrum of L-Cys.

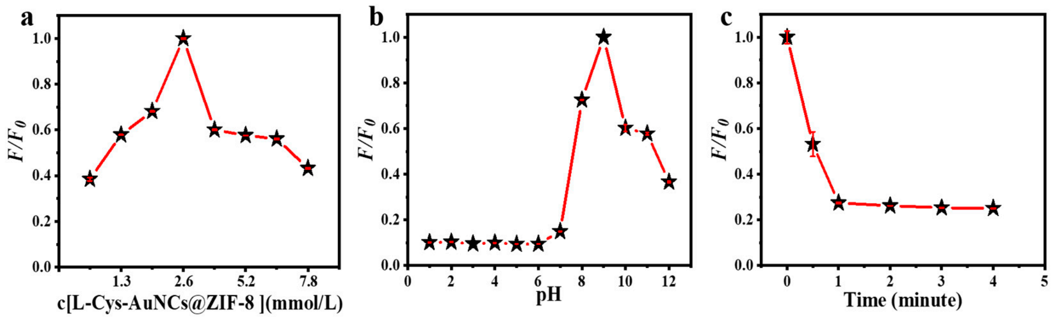

Figure S5. Optimization of L-Cys-AuNCs@ZIF-8 concentration (a); pH (b); and reaction time (c) with addition of copper ions alone.

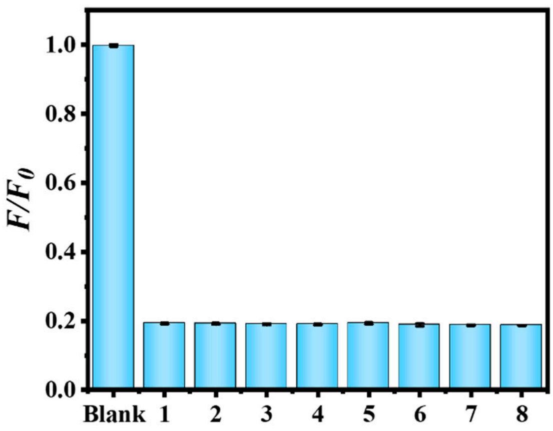

Figure S6. Reproducibility of Cu^{2+} detection.
